# A propensity score matching study on clinical characteristics and risk factors of herpes zoster in malignant tumor

**DOI:** 10.3389/fonc.2026.1804448

**Published:** 2026-05-13

**Authors:** Yang Li, Canhua Liang, Ziwei Feng, Shaohuan Lu, GuangZhao Wang, Guangyi Meng

**Affiliations:** Department of Pharmacy, The First People’s Hospital of Yulin, Yulin, Guangxi, China

**Keywords:** factor influencing, hematologic cancers, herpes zoster, malignant tumor, propensity score matching

## Abstract

**Background:**

Herpes zoster (HZ) is caused by the reactivation of latent varicella-zoster virus (VZV). Cancer patients, due to immune suppression, are at an increased risk of HZ. However, the exact relationship between cancer and the occurrence of HZ remains unclear.

**Methods:**

This retrospective study collected data from cancer patients diagnosed between 01/01/2020, and 30/09/2024. Descriptive statistics were used to summarize the clinical characteristics of HZ patients. Propensity score matching (PSM) was applied to match HZ and non-HZ groups in a 1:3 ratio, controlling for potential confounding factors. Logistic regression analysis was performed to investigate the impact of cancer type, treatment modalities, and other factors on the occurrence of HZ.

**Results:**

Among 99 HZ patients, the onset of HZ was closely associated with the timing of anticancer treatment, with 89.89% of patients developing HZ within 12 months after the first anticancer therapy. After PSM matching, cancer type was identified as a significant independent risk factor for HZ. Patients with hematologic cancers, such as multiple myeloma, had a significantly higher risk of developing HZ compared to those with solid tumors (OR = 2.41, 95% CI 1.11-5.24, P = 0.03).

**Conclusions:**

Hematologic malignancy patients are at a significantly higher risk of developing HZ compared to those with solid tumors. The use of immune checkpoint inhibitors (ICIs) did not significantly increase the risk of HZ. These findings suggest that patients with hematologic cancers should be closely monitored for HZ and undergo preventive measures.

## 1 Introduction

Herpes Zoster (HZ) is caused by the reactivation of latent varicella-zoster virus (VZV), which results in clinical manifestations such as erythema, blisters, and other skin symptoms, often accompanied by neuralgia. In severe cases, it can lead to skin ulcers or secondary infections (1). During the primary VZV infection, the virus may be transported retrogradely along the nerve axons from the infected skin. Alternatively, the virus may be carried via T cells into the sensory neurons of the dorsal root ganglion, where it remains latent (2). When the immune system is weakened—due to factors such as aging, excessive fatigue, psychological stress, HIV, immunosuppressive therapy, or malignancy—the latent VZV may be reactivated, traveling along the nerve axons to infect the skin cells in the region supplied by that nerve, initiating an inflammatory response (3). Common complications of HZ include neuralgia, nerve paralysis, and secondary infections, which significantly affect the patient’s quality of life (4). Research on HZ is crucial in improving the quality of survival of cancer patients.

Cancer patients often experience immune dysfunction, which may be exacerbated by chemotherapy, psychological stress, surgery, or radiotherapy, leading to cytokine-mediated immune suppression. These factors collectively increase the risk of HZ in cancer patients (5, 6). Additionally, the interaction between viral infections, the human immune system, and cancer biology is highly complex. A large prospective cohort study conducted in Australia by Qian et al. from 2006 to 2016, involving over 240, 000 adults, found that cancer patients had a significantly higher incidence of HZ after the diagnosis of cancer, compared to those without cancer (7).In a 2017 study, 192, 081 adults with HZ (compared to 732, 035 controls) were analyzed to explore the risk of developing HZ following certain cancers. The results revealed a positive correlation between cancer and the risk of HZ (adjusted OR 1.29), with hematological cancers (multiple myeloma, lymphoma, and leukemia) showing a stronger association with HZ (OR 2.46). Among solid tumors, the central nervous system cancers had the strongest association with HZ (OR 2.31) (8).To date, the precise relationship between cancer diagnosis and HZ incidence has not been firmly established. The existing literature, as well as our observations of HZ cases in clinical practice, have prompted us to investigate the factors influencing the development of HZ in cancer patients.

This study utilized real-world data to provide a descriptive analysis of the occurrence of HZ among cancer patients in a Chinese population, and to explore the impact of immune checkpoint inhibitors (ICIs) and other factors on the risk of HZ. We hope that our findings will assist clinicians in better identifying high-risk populations for HZ among cancer patients.

## 2 Materials and methods

### 2.1 Study population

This retrospective study collected clinical data from cancer patients diagnosed between 01/01/2020, and 30/09/2024. The collected data included: age, sex, BMI, underlying diseases, tumor type, smoking history, alcohol history, anticancer therapy, use of ICIs, and history of tumor surgery. Based on whether HZ was diagnosed after the cancer diagnosis, patients were divided into two groups: the non-HZ group and the HZ group. The inclusion criteria were as follows: 1) Confirmed diagnosis of malignant tumors based on clinical presentation, imaging studies, and histopathological examination. 2) The first diagnosis of HZ in accordance with the “Chinese Expert Consensus on the Diagnosis and Treatment of Herpes Zoster (2022 Edition).” Exclusion criteria: Incomplete clinical data, including missing BMI, underlying diseases, or other basic information. This study has been reviewed by the Research Ethics Committee of The First People’s Hospital of Yulin, which confirmed that ethical approval is not required (YLSY-IRB-SR-2026004). This is because it is a retrospective analysis of existing patient data and does not involve any harm to patients or the use of human tissue samples. Additionally, the data used could not identify individual participants during or after data collection.

### 2.2 Study methods

To investigate the occurrence of HZ, descriptive statistics were performed on the location of HZ lesions, onset time, cancer duration, and the time interval between the first anticancer treatment and HZ onset. To control for potential confounding factors, propensity score matching (PSM) was used to match patients in the non-HZ and HZ groups. The matching was performed using 12 covariates: age, sex, BMI, underlying diseases (hypertension, diabetes, coronary heart disease), tumor type, smoking history, alcohol history, anticancer therapy, use of ICIs, and history of tumor surgery, with a 1:3 ratio. It is noteworthy that the study included a wide range of tumor patients, involving multiple types of chemotherapeutic agents and combination regimens, thus chemotherapy was considered as a single variable. The matching was done using a caliper matching method without replacement, with a caliper value set at 0.1. Standardized mean differences (SMD) were used to assess the balance between the groups after matching, with SMD < 0.1 indicating good balance. After matching, logistic regression was performed with HZ occurrence as the dependent variable to compare the two groups. E-values were calculated to assess the robustness of the results to unmeasured confounding.

### 2.3 Statistical analysis

Data collection, organization, and statistical analysis were performed using SPSS 26.0 software, with a two-tailed significance level of α = 0.05. For continuous variables, normally distributed data were described using mean ± standard deviation (x ± s), while skewed data were described using median (M) and interquartile range (P_25_, P_75_). Between-group comparisons were made using t-tests or rank-sum tests. For categorical variables, frequencies and percentages were reported, and comparisons between groups were conducted using chi-square (χ^2^) tests. After completing PSM (R4.4.2), logistic regression analysis was conducted to further adjust for potential residual confounding and to identify influencing factors. Odds ratios (ORs) with 95% confidence intervals (CIs) were calculated, and statistical significance was defined as P < 0.05.

## 3 Results

### 3.1 Herpes zoster incidence

**Figure 1:**
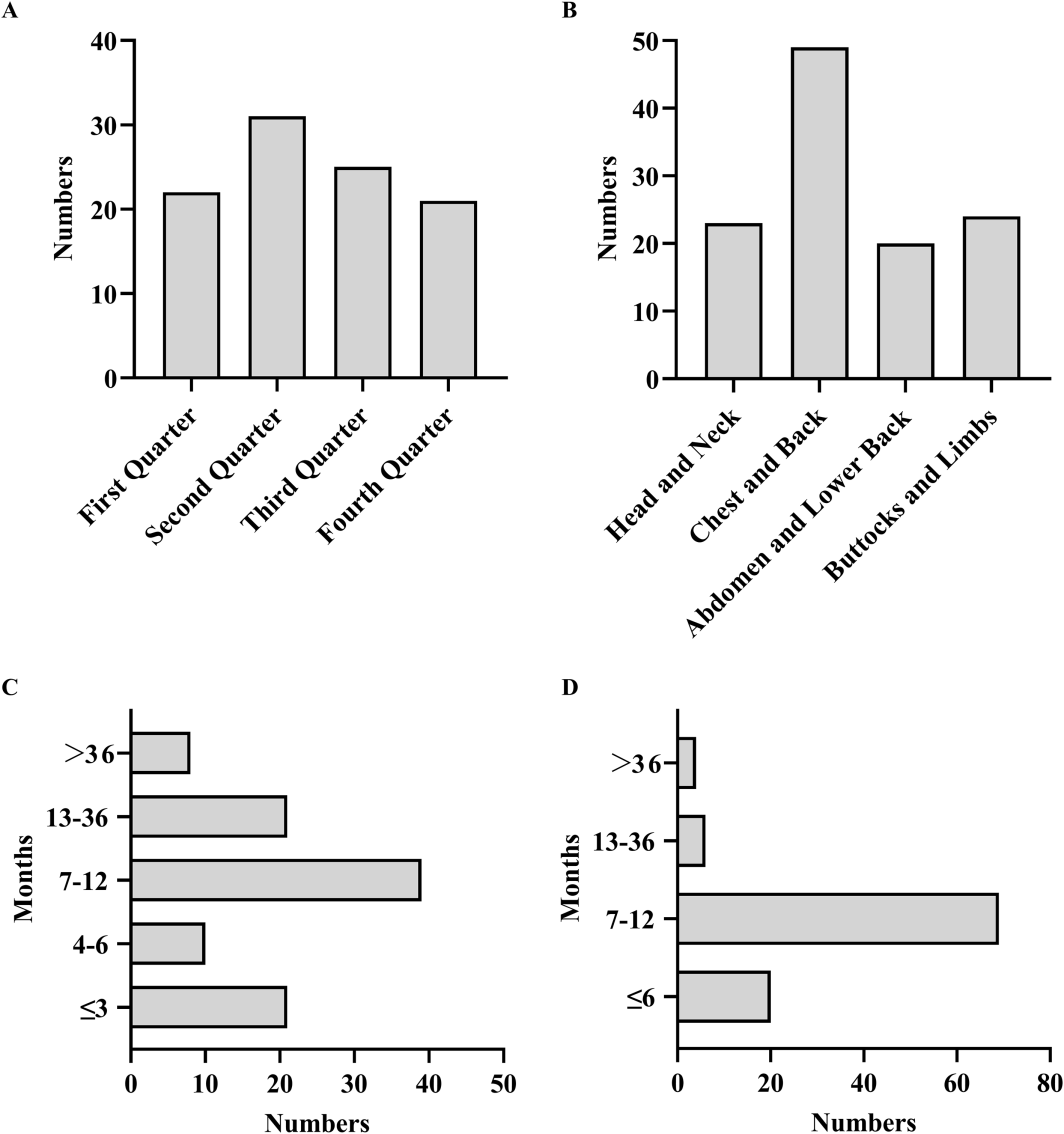
Clinical characteristic of herpes zoster. **(A)** Temporal Distribution of HZ; **(B)** Anatomical Distribution of HZ; **(C)** Time Interval Between HZ Onset and Tumor Diagnosis; **(D)** Time Interval Between HZ Onset and First Antineoplastic Therapy.

A total of 99 HZ patients were included in this study. As shown in **Figure 1A**, the distribution of HZ cases by onset month is as follows: 22 cases in the first quarter (January to March), 31 cases in the second quarter (April to June), 25 cases in the third quarter (July to September), and 20 cases in the fourth quarter (October to December). This indicates a relatively even distribution of cases throughout the year. The most common affected areas were the chest and back, with 15 patients presenting with involvement in ≥2 regions (**Figure 1B**). Among the patients, 41.41% had a cancer duration of 7–12 months, with the average cancer duration being 2.85 months (**Figure 1C**). Furthermore, we analyzed the time interval between the first anticancer treatment and the onset of HZ. Among the patients, 89 (89.89%) developed HZ within 12 months of the first anticancer treatment, while 4 patients developed HZ more than 36 months after start of therapy (**Figure 1D**).

### 3.2 Comparison of baseline characteristics before and after propensity score matching

**Figure 2:**
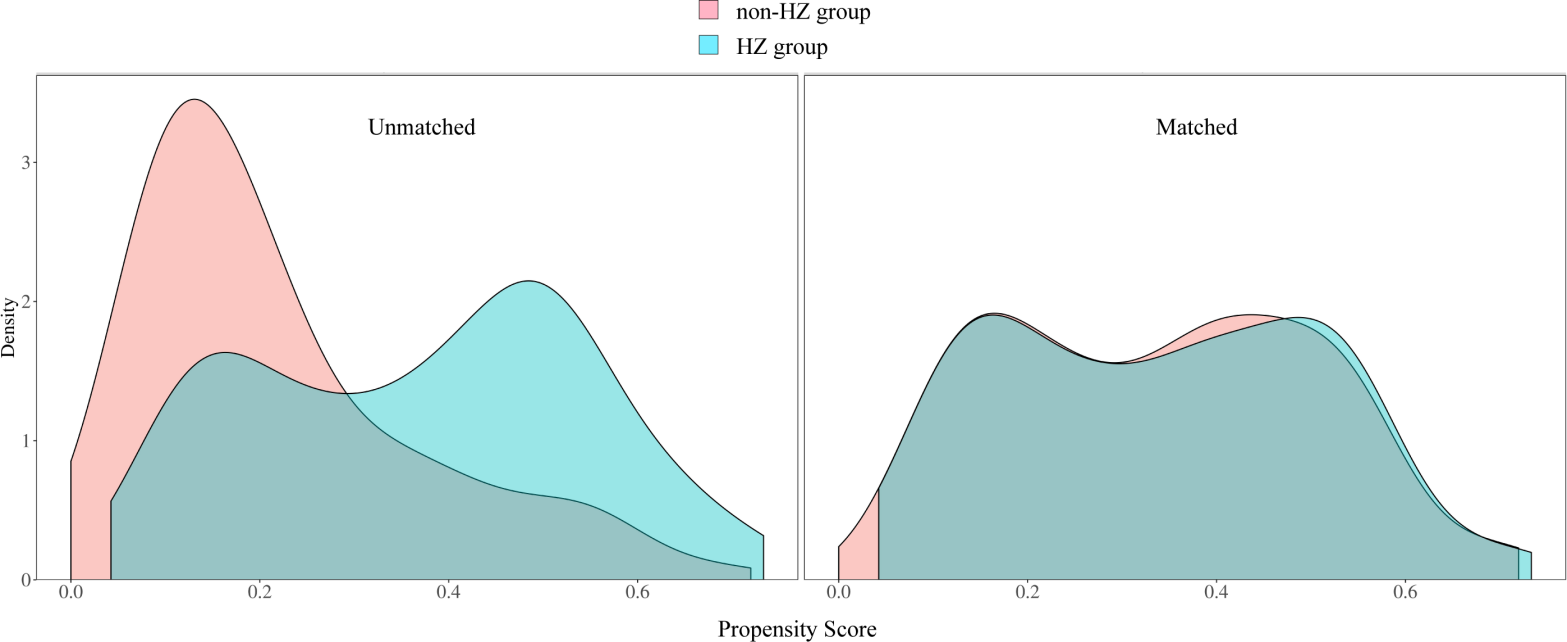
Propensity score distribution.

**Figure 3:**
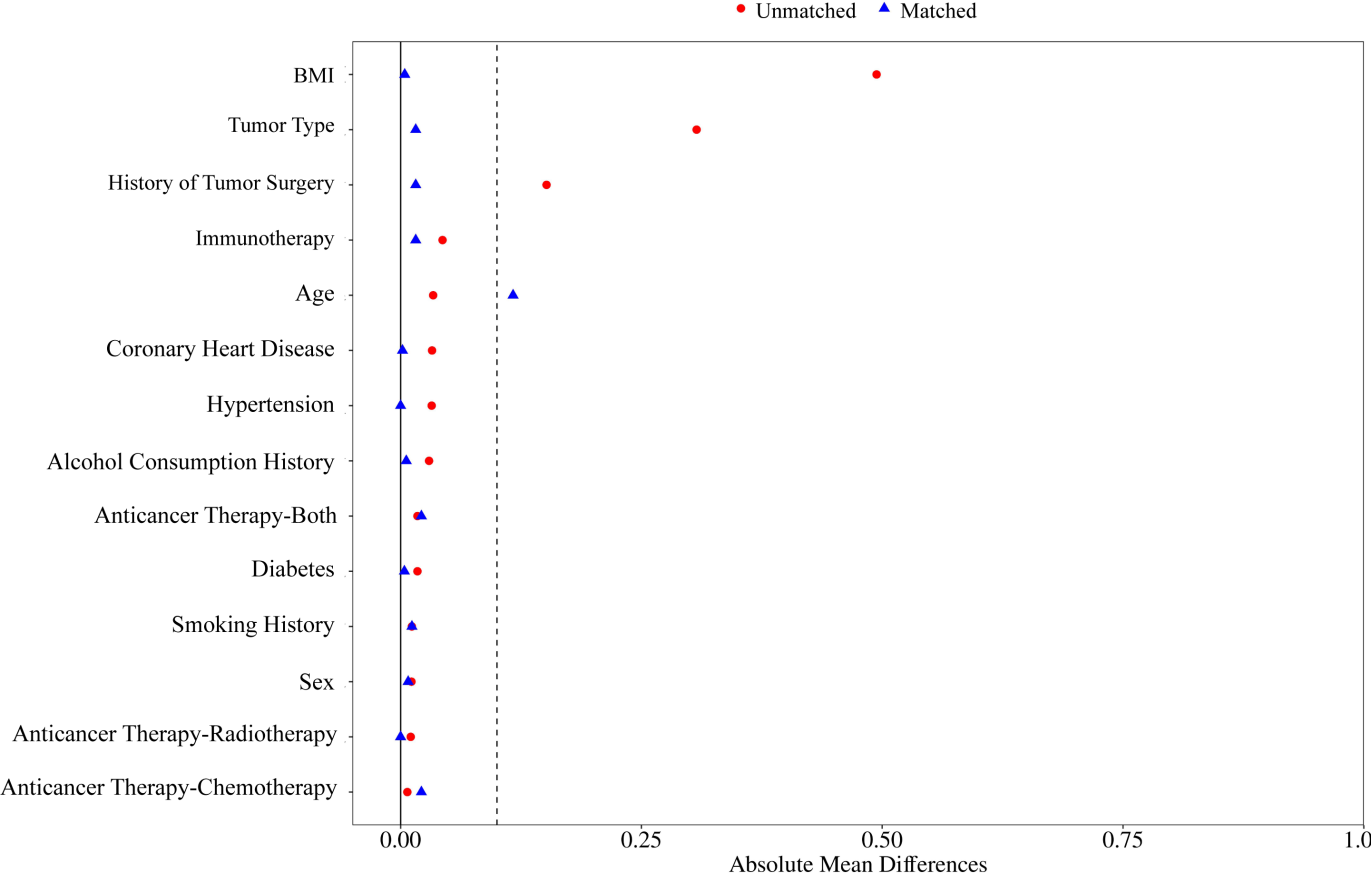
Absolute mean differences comparing baseline characteristics between the two groups.

According to the inclusion and exclusion criteria, the non-HZ group included 286 patients, and the HZ group included 99 patients. After PSM, 85 patients from the HZ group and 177 patients from the non-HZ group were included, achieving a matching success rate of 85.86%. This indicates a well-balanced matching process between the two groups. Before matching, significant differences were observed between the two groups in terms of BMI, cancer type, and cancer surgery history, with multiple covariates having standardized mean differences (SMD) > 0.1, lack of comparability. After matching, only smoking history and alcohol history had SMDs slightly > 0.1, while the remaining variables had SMDs < 0.1. The P-values for all variables were ≥ 0.05, confirming that the baseline characteristics were well-balanced between the two groups after matching (**Table 1**). The propensity score distributions for the HZ and non-HZ groups were highly overlapped, with similar peak values and widths, suggesting good balance in baseline characteristics, as shown in **Figure 2**. In the balance test, the standardized biases for all covariates decreased after PSM, achieving an optimal matching effect (**Figure 3**).

**Table 1:** Baseline characteristics of two groups of patients before and after matching.

Variable	Before PSM(n=385)				After PSM (n = 262)			
	Non-HZ (n = 286)	HZ (n = 99)	*P*	SMD	Non-HZ (n = 177)	HZ (n = 85)	*P*	SMD
Age, M (Q_25_, Q75)	58.00 (52.00, 66.00)	61.00 (51.00, 68.00)	0.546	-0.034	59.00 (53.00, 66.00)	60.00 (50.00, 68.00)	0.821	-0.094
Sex, n (%)			0.847	0.023			0.772	-0.038
Male	147 (51.40)	52 (52.53)			95 (53.67)	44 (51.76)		
Female	139 (48.60)	47 (47.47)			82 (46.33)	41 (48.24)		
BMI, Mean ± SD	22.44 ± 3.70	20.92 ± 3.09	<.001	-0.494	21.68 ± 3.36	21.23 ± 3.14	0.299	-0.144
Tumor Type, n (%)			<.001	-0.618			0.05	-0.254
Solid tumor	215 (75.17)	44 (44.44)			112 (63.28)	43 (50.59)		
Blood Cancer	71 (24.83)	55 (55.56)			65 (36.72)	42 (49.41)		
Hypertension, n (%)			0.51	-0.074			0.78	-0.036
No	223 (77.97)	74 (74.75)			134 (75.71)	63 (74.12)		
Yes	63 (22.03)	25 (25.25)			43 (24.29)	22 (25.88)		
Diabetes, n (%)			0.576	-0.061			0.96	0.007
No	265 (92.66)	90 (90.91)			160 (90.40)	77 (90.59)		
Yes	21 (7.34)	9 (9.09)			17 (9.60)	8 (9.41)		
Coronary Heart Disease, n (%)			0.237	-0.137			0.864	-0.058
No	278 (97.20)	93 (93.94)			169 (95.48)	80 (94.12)		
Yes	8 (2.80)	6 (6.06)			8 (4.52)	5 (5.88)		
Smoking History, n (%)			0.775	-0.033			0.422	0.114
No	246 (86.01)	84 (84.85)			145 (81.92)	73 (85.88)		
Yes	40 (13.99)	15 (15.15)			32 (18.08)	12 (14.12)		
Alcohol Consumption History, n (%)			0.295	0.15			0.417	0.125
No	266 (93.01)	95 (95.96)			164 (92.66)	81 (95.29)		
Yes	20 (6.99)	4 (4.04)			13 (7.34)	4 (4.71)		
History of Tumor Surgery, n (%)			0.006	0.371			0.524	0.087
No	182 (63.64)	78 (78.79)			131 (74.01)	66 (77.65)		
Yes	104 (36.36)	21 (21.21)			46 (25.99)	19 (22.35)		
Anticancer Therapy, n (%)			0.701	-0.018			0.966	-0.006
Chemotherapy	236 (82.52)	81 (81.82)			142 (80.23)	68 (80.00)		
Radiotherapy	3 (1.05)	0 (0.00)			0 (0.00)	0 (0.00)		
Both	47 (16.43)	18 (18.18)			35 (19.77)	17 (20.00)		
Immunotherapy, n (%)			0.313	0.129			0.735	0.046
No	236 (82.52)	86 (86.87)			147 (83.05)	72 (84.71)		
Yes	50 (17.48)	13 (13.13)			30 (16.95)	13 (15.29)		

PSM, Propensity score matching; SMD, Standardized mean differences.

### 3.3 Factors influencing the occurrence of HZ

After matching the clinical data, univariate analysis was conducted to identify factors influencing HZ occurrence. Among all the variables, only cancer type showed a significant association with HZ. Compared to patients with solid tumors, patients with hematological malignancies had a significantly higher risk of HZ (OR = 1.68, P = 0.049). Among hematological malignancies, patients with lymphoma had a significantly lower risk of the outcome than those with multiple myeloma, while there was no statistically significant difference in risk between acute leukemia and multiple myeloma. After adjusting for other confounding factors, this association remained significant and the effect size increased, with the risk of the outcome in patients with hematological malignancies rising to 2.41 times that of patients with solid tumors (P = 0.030). The E-value was 4.25, indicating that an unmeasured confounder would need to be associated with both hematological malignancy and HZ by a risk ratio of 4.25 or more to fully explain away the observed association. Regarding the comparison among hematological malignancies, the risk of developing HZ in patients with lymphoma was only 11.7% of that in patients with multiple myeloma (P = 0.010), and there remained no significant difference between acute leukemia and multiple myeloma (**Table 2**).

**Table 2:** Logistic regression analysis in two groups of patients after matching.

Variables	Univariable analysis				Multivariable analysis			
	β	SE	*P*	OR (95%CI)	β	SE	*P*	OR (95%CI)
Age	-0.01	0.01	0.42	0.99 (0.97 - 1.01)	-0.01	0.01	0.25	0.99 (0.97 - 1.01)
Sex	0.08	0.26	0.77	1.08 (0.64 - 1.81)	-0.1	0.31	0.75	0.91 (0.49 - 1.67)
Male				1.00 (Reference)				1.00 (Reference)
Female								
BMI	-0.04	0.04	0.3	0.96 (0.89 - 1.04)	-0.06	0.04	0.2	0.95 (0.87 - 1.03)
Tumor Type								
Solid tumor				1.00 (Reference)				1.00 (Reference)
Blood Cancer	0.52	0.27	0.049	1.68 (1.01 - 2.84)	0.88	0.4	0.03	2.41 (1.11 - 5.24)
Multiple Myeloma				1.00 (Reference)				1.00 (Reference)
Lymphoma			0.003	0.12 (0.03 - 0.47)			0.010	0.12 (0.02 - 0.59)
Acute leukemia			0.617	0.67 (0.14 - 3.27)			0.787	0.78 (0.13 - 4.59)
Hypertension	0.08	0.3	0.78	1.09 (0.60 - 1.97)	0.12	0.34	0.72	1.13 (0.58 - 2.21)
No				1.00 (Reference)				1.00 (Reference)
Yes								
Coronary Heart Disease	0.28	0.59	0.64	1.32 (0.42 - 4.16)	0.03	0.63	0.96	1.04 (0.30 - 3.53)
No				1.00 (Reference)				1.00 (Reference)
Yes								
Diabetes	-0.02	0.45	0.96	0.98 (0.40 - 2.37)	0.16	0.48	0.74	1.17 (0.46 - 3.02)
No				1.00 (Reference)				1.00 (Reference)
Yes								
Smoking History	-0.29	0.37	0.42	0.74 (0.36 - 1.53)	-0.07	0.47	0.89	0.94 (0.37 - 2.34)
No				1.00 (Reference)				1.00 (Reference)
Yes								
Alcohol Consumption History	-0.47	0.59	0.42	0.62 (0.20 - 1.97)	-0.31	0.7	0.65	0.73 (0.19 - 2.86)
No				1.00 (Reference)				1.00 (Reference)
Yes								
History of Tumor Surgery	-0.2	0.31	0.52	0.82 (0.45 - 1.51)	0.18	0.39	0.65	1.19 (0.56 - 2.54)
No				1.00 (Reference)				1.00 (Reference)
Yes								
Anticancer Therapy	0.01	0.33	0.97	1.01 (0.53 - 1.94)	0.32	0.39	0.4	1.38 (0.65 - 2.95)
Chemotherapy				1.00 (Reference)				1.00 (Reference)
Both								
Immunotherapy	-0.12	0.36	0.74	0.88 (0.44 - 1.80)	0.15	0.43	0.73	1.16 (0.50 - 2.67)
No				1.00 (Reference)				1.00 (Reference)
Yes								

OR, Odds Ratio; CI, Confidence Interval.

## 4 Discussion

In this study, we identified cancer type as a significant risk factor for the development of HZ in cancer patients. Compared to patients with solid tumors, those with hematological cancers were at a significantly higher risk of developing HZ. Our findings are consistent with previous studies. For instance, a large study conducted in the UK included 192, 081 HZ patients and 732, 035 controls, and found a positive correlation between cancer diagnosis and the incidence of HZ (adjusted OR 1.29, 95% CI 1.27-1.32). This association was particularly strong in patients with hematological cancers, with an OR of 2.46 (95% CI 2.33-2.60) (8). This increased risk may be attributed to the nature of hematological cancers or factors like high-intensity chemotherapy and stem cell transplantation. In our cohort, most patients with hematological cancers were diagnosed with multiple myeloma (MM), which may partly explain the increased risk of HZ. For MM, dysregulation of plasma cell activity weakens the patient’s immune defenses, and long-term use of steroids and proteasome inhibitors is a key contributing factor to the development of HZ (9). Proteasome inhibitors are widely used in MM treatment, effective in both previously untreated and relapsed/refractory cases (10, 11). Several studies have shown that patients with MM treated with bortezomib have an increased risk of developing HZ (12–14). In the phase III APEX trial, the risk of HZ in patients with relapsed MM was even higher in those receiving bortezomib compared to those treated with high-dose dexamethasone (15). Given the risk of HZ, a key question is whether cancer patients should receive preventive antiviral treatment. Research indicates that prophylactic antiviral treatment is beneficial for preventing HZ in MM patients, with acyclovir dosages ranging from 200 mg once daily to 400 mg three times daily (16, 17). Some studies suggest that intermittent antiviral therapy results in a similar incidence of HZ compared to continuous treatment, with no significant safety difference. Intermittent prophylactic measures can reduce the duration and frequency of medication, thereby extending drug-free periods and improving patient adherence to treatment (16). The National Comprehensive Cancer Network (NCCN) recommends that all MM patients undergoing proteasome inhibitor therapy should receive HZ prophylaxis, although the optimal duration of preventive treatment and the need for HZ prevention for other treatments beyond proteasome inhibitors remains to be further evaluated. Based on our findings, we suggest that patients with multiple myeloma receiving proteasome inhibitor therapy should routinely receive antiviral prophylaxis. Intermittent dosing strategies may also be considered according to patient tolerance, in order to maintain preventive efficacy while improving long-term treatment adherence. For patients with other types of malignancies, close clinical monitoring remains the preferred management approach. In the field of cancer therapy, ICIs have become an important treatment modality, and their potential impact on the risk of HZ has attracted increasing attention. ICIs restore and enhance antitumor immunity by blocking inhibitory pathways such as PD-1/PD-L1 and promoting the function of effector T cells (18). Based on this mechanism, ICIs are generally not considered to cause traditional immunosuppression and, theoretically, should not significantly increase the risk of opportunistic infections. However, emerging evidence from clinical practice suggests that this effect may be more complex. For example, cases of tuberculosis have been reported in patients receiving nivolumab (19), indicating that ICIs may exert bidirectional or context-dependent immunomodulatory effects rather than simply enhancing immune function. In addition, case reports have described severe viral infections following ICI therapy. Watanabe et al. reported a case of VZV encephalitis in a lung cancer patient occurring five days after the 31st cycle of nivolumab monotherapy (20). Similarly, Gozzi et al. reported a case of HZ in a patient with metastatic lung adenocarcinoma during nivolumab treatment (21). Furthermore, observational studies have suggested a potential association between ICIs and increased HZ risk. Yoshiaki et al. and Qiao et al. both reported a higher incidence of HZ in lung cancer patients treated with PD-1/PD-L1 inhibitors (22, 23). Taken together, these findings suggest that ICIs may alter immune homeostasis and potentially trigger the reactivation of latent viruses, particularly during phases of immune reconstitution. This phenomenon may be related to immune reconstitution inflammatory syndrome (24). In addition, another potential risk factor for HZ during immunotherapy is the use of corticosteroids for the management of immune-related adverse events, which may exert direct inhibitory effects on T-cell function (25). However, in the present study, no statistically significant association was observed between ICI use and the risk of HZ. This finding should be interpreted with caution. The relatively small sample size, particularly the limited number of patients in the ICI subgroup, may have reduced the statistical power to detect modest associations. Moreover, as a retrospective study, residual confounding factors—such as lines of therapy, combination treatment regimens, and dynamic changes in immune status—could not be fully accounted for. Therefore, our results do not exclude a potential association between ICIs and HZ. Larger, well-designed prospective studies are warranted to further clarify the impact of ICIs on both the risk and timing of HZ occurrence. HZ not only causes significant neuropathic pain but may also lead to viral transmission or secondary bacterial infections, thereby impairing quality of life and potentially delaying anticancer treatment. Therefore, in line with the American Society of Clinical Oncology (ASCO) guidelines, it is advisable to assess the need for VZV screening prior to initiating PD-1/PD-L1 inhibitor therapy (26). In addition, vaccination may be considered 2–3 weeks before the initiation of ICI therapy (27).

Our study focused on cancer patients who developed HZ after their cancer diagnosis. However, literature suggests that the relationship between cancer and HZ may not be one-directional. The potential association between HZ and subsequent cancer risk is also worth attention. A meta-analysis, which included 46 studies, attempted to explore the relationship between HZ and cancer. The study found that the relative risk of developing cancer after a diagnosis of HZ was 1.42 (95% confidence interval: 1.18, 1.71) overall, and 1.83 (95% confidence interval: 1.17, 2.87) one year after the onset of HZ. The highest estimates were generally reported for occult hematological cancers. The absolute risk of any cancer one year after presenting with HZ ranged from 0.7% to 1.8% (28). Another retrospective cohort study found that patients diagnosed with HZ ophthalmicus had a 9.25-fold (95% confidence interval: 5.51-15.55) increased risk of subsequent cancer compared to a matched control group. Furthermore, the 1-year cancer-free survival rates in HZ ophthalmicus patients were significantly lower than those in the control group (29). Although the exact mechanisms underlying the increased cancer risk after HZ diagnosis remain unclear, we speculate that it may be related to a decline in the immune surveillance function of cancer cells after immune system disruption and the reactivation of the virus. Moreover, various herpesviruses are known to directly contribute to cancer development through pro-inflammatory effects, immune evasion, and immune suppression (30, 31). Another possible explanation is that some occult cancers, which have not yet shown clear clinical symptoms during the early stages of cancer cell proliferation, still cause immune system damage, and thus the diagnosis of HZ may precede the diagnosis of cancer by months or even years. However, some studies have challenged the conclusion that a diagnosis of HZ increases cancer risk. A study published early on in *The New England Journal of Medicine* followed 590 patients and found that the overall relative risk of developing cancer after a diagnosis of HZ was 1.1 (95% confidence interval: 0.9 to 1.3). Except for slightly higher relative risks for colon and bladder cancers in women, no significant differences were observed in other cancer sites. Therefore, this study did not support enhanced cancer monitoring for patients diagnosed with HZ (32).Given the current body of evidence, it is difficult to establish a causal relationship between HZ and cancer. There is insufficient evidence to suggest whether patients with HZ should undergo cancer screening. However, it appears necessary to monitor HZ in cancer patients who are already diagnosed.

Given that 89.89% of patients in this study developed HZ within 12 months after the initiation of treatment, this period may represent a high-risk window for HZ occurrence. During this time, enhanced patient education and closer clinical monitoring may be warranted.

This study has several limitations. First, in terms of methodological design, while we employed PSM to minimize inherent selection bias and enhance internal validity, achieving a rigorous covariate balance necessitated the exclusion of approximately 14.14% of cases. This reduction in sample size may introduce a new degree of selection bias. Second, limited by the characteristics of the retrospective database, some key clinical information could not be included in the model. Since information on herpes zoster vaccination and prophylactic antiviral medication is not standardized data mandatorily collected upon admission, such information was difficult to trace in the retrospective analysis. Meanwhile, considering the widespread use of corticosteroids in this study cohort, including them as a single covariate in the model might introduce bias. Laboratory indicators with significant dynamic fluctuations, such as white blood cell and lymphocyte counts, were also not included as covariates to avoid potential bias resulting from the extraction of baseline data only. Finally, as cancer patients in real-world clinical treatment often receive complex combination regimens with highly overlapping drug effects, forcibly subdividing chemotherapy drugs into subgroups would severely weaken statistical power and potentially lead to model overfitting. Therefore, we treated chemotherapy as a binary variable; while this ensures the robustness of the model, it may also mask the specific risks associated with particular drugs. Future large-scale, multicenter prospective studies are still needed to validate these observations and further refine the risk stratification of HZ in cancer patients.

## 5 Conclusion

Our study demonstrates that cancer type is an important risk factor for the occurrence of HZ in cancer patients. Compared to solid tumors, hematological cancers are associated with a significantly higher risk of developing HZ. The use of ICIs does not appear to affect the incidence of HZ.

## Data Availability

Publicly available datasets were analyzed in this study. This data can be found here: Not applicable.
